# Towards refactoring the Molecular Function Ontology with a UML profile for function modeling

**DOI:** 10.1186/s13326-017-0152-y

**Published:** 2017-10-04

**Authors:** Patryk Burek, Frank Loebe, Heinrich Herre

**Affiliations:** 1 Institute of Medical Informatics, Statistics and Epidemiology, University of Leipzig, Haertelstrasse 16-18, Leipzig, 04107 Germany; 2 Computer Science Institute, University of Leipzig, Augustusplatz 10, Leipzig, 04109 Germany

**Keywords:** Gene Ontology, Molecular Function Ontology, Unified Modeling Language, Ontology, Function decomposition, Intensional subsumption

## Abstract

**Background:**

Gene Ontology (GO) is the largest resource for cataloging gene products. This resource grows steadily and, naturally, this growth raises issues regarding the structure of the ontology. Moreover, modeling and refactoring large ontologies such as GO is generally far from being simple, as a whole as well as when focusing on certain aspects or fragments. It seems that human-friendly graphical modeling languages such as the Unified Modeling Language (UML) could be helpful in connection with these tasks.

**Results:**

We investigate the use of UML for making the structural organization of the Molecular Function Ontology (MFO), a sub-ontology of GO, more explicit. More precisely, we present a UML dialect, called the **Fu**nction Mod**e**ling **L**anguage (FueL), which is suited for capturing functions in an ontologically founded way. FueL is equipped, among other features, with language elements that arise from studying patterns of subsumption between functions. We show how to use this UML dialect for capturing the structure of molecular functions. Furthermore, we propose and discuss some refactoring options concerning fragments of MFO.

**Conclusions:**

FueL enables the systematic, graphical representation of functions and their interrelations, including making information explicit that is currently either implicit in MFO or is mainly captured in textual descriptions. Moreover, the considered subsumption patterns lend themselves to the methodical analysis of refactoring options with respect to MFO. On this basis we argue that the approach can increase the comprehensibility of the structure of MFO for humans and can support communication, for example, during revision and further development.

## Background

Gene Ontology (GO) [1, 2] is an important, widely used, very large and continuously growing resource for cataloging gene products. In 2000 GO contained less than 5000 terms, which increased to circa 13,000 in 2003 [1], exceeded 30,000 in 2010 [3] and is close to 45,000 terms in July 2017 [2]. The Molecular Function Ontology (MFO) is a sub-ontology of GO of more than 11,000 terms in 2017. This growth of the ontology leads to a suboptimal structure [3]. Clearly, the GO Consortium itself is constantly improving and evolving GO. In addition, size and importance of the ontology and the recognition of problems have motivated refactoring initiatives, see [4, 5], for example. Overall, it turns out that modeling and refactoring large ontologies such as GO are difficult tasks, which should be supported by human-friendly representations. Serialization formats used for machine processing of ontologies, such as the OBO flat file format [6] or the Web Ontology Language (OWL) [7], are not the easiest to be used by humans. This motivates proposing the adoption of human-friendly graphical notations for certain purposes, like languages used in software engineering, already employed for the task of ontology representation [8, 9].

The Unified Modeling Language (UML) [10, 11], developed and maintained by the Object Management Group (OMG) [12], is the de facto standard for graphical conceptual modeling of software systems. Moreover, UML has a high potential for various applications that go beyond software engineering, among them modeling biological knowledge and biological ontologies [4, 13], for several reasons. First, there is a rich infrastructure. Numerous tools for UML modeling are available on the market and can be used out of the box for visualizing biological ontologies as a whole or in part. Another advantage is its adaptability. UML is equipped with extension mechanisms such as stereotypes and profiles, which support the easy construction of domain- or task-specific UML dialects. For example, a UML profile for the OBO relations ontology is proposed in [4].

In the present paper we investigate if UML, more precisely, a dedicated dialect, can be utilized for making the structure of the Molecular Function Ontology more explicit and if it can support the refactoring of MFO. The focus on MFO within GO results from having dealt with the notion of function from a general point of view in earlier work, e.g. [14]. The “Methods” section establishes foundations by sketching some features of functions in MFO, describing an intensional understanding of subsumption, and introducing the **Fu**nction Mod**e**ling **L**anguage (FueL) as a UML dialect that is suited for function modeling. Concerning results, section “Modeling molecular functions with FueL” introduces core elements of FueL that are required to analyze the subsumption patterns that section “Patterns of function subsumption” defines based on FueL. Then we are prepared to illustrate the application of FueL to MFO in the “Application” section by modeling the structure of molecular functions and proposing some refactoring options. The “Discussion” section is mainly devoted to related work and to the applicability of FueL at this stage. It further indicates directions of future work, before the paper ends with section “Conclusions”.

## Methods

### Molecular Function Ontology

Like all GO terms, functions in MFO are specified by id, name, natural language definition and an optional list of synonyms. For instance, the function of catalyzing carbohydrate transmembrane transport is specified by id: GO:0015144; name: *carbohydrate transmembrane transporter activity*; definition: catalysis of the transfer of carbohydrate from one side of the membrane to the other; synonym: sugar transporter. Additionally, for each function its relations with other concepts can be captured. The semantics of the relations that are used for this purpose is provided by serialization languages such as the OBO flat file format or OWL, and/or by the OBO relations ontology (RO) [15]. In particular, functions in MFO are organized into a hierarchy by means of the is_a link from RO; furthermore, they are linked with processes by the part_of relationship from RO; and in some cases they have relations with concepts of other ontologies such as ChEBI [16]. For instance, GO:0015144 is linked, by means of the RO is_a relation, to its parent functions GO:1901476 *carbohydrate transporter activity* and GO:0022891 *substrate-specific transmembrane transporter activity*, by means of the RO part_of relation to the process GO:0034219: *carbohydrate transmembrane transport*, and by means of the RO transports_or_maintains_localization_of to CHEBI:16646: *carbohydrate*.

From the above we see that the semantics of functions in MFO is provided to a large extent by informal natural language expressions and partially by relations with other concepts.

### Intensional subsumption

We propose defining the notion of function subsumption, which is a backbone of MFO, upon an intensional interpretation of the is_a relation. Typically, in the field of ontology engineering the extensional aspect of the is_a relation is stressed; in OWL, for instance, A is a subclass of B if every instance of A is an instance of B. The same interpretation is used in RO, where is_a is defined by the reference to the sets of all instances (extensions) of the concepts. According to this understanding the is_a relation is often called extensional subsumption, in contrast to its intensional counterpart(s), where we focus on structural subsumption [17].

Instead of referring to instances, structural subsumption is defined based on the structure of a concept. The latter can be understood as a composition of conceptual parts by means of various composing relations. For illustration within GO itself, GO:0005215: *transporter activity* is justified to intensionally subsume GO:0022857: *transmembrane transporter activity*, because, following [17], both are activities and they are (partially) defined by part_of relations to GO:0006810: *transport* and to GO:0055085: *transmembrane transport*, resp., and the latter is subsumed by the former. Overall, the main assumption is that concepts are complex structures which can be organized into a subsumption hierarchy. The reading of intensional subsumption is similar to inheritance in object-oriented languages, where one class inherits its structure from another. That enables the structuring of classes into hierarchies. Note that extensional and intensional subsumption need not be seen to be in conflict with each other, but they can be understood as different facets of the hierarchical organization of classes.

### UML, UML profiles and FueL

The Unified Modeling Language (UML) [10, 11] is a rich graphical modeling language developed originally for the support of software engineering. Currently, its applications go beyond software engineering, covering a broad spectrum of domains, including systems and enterprise modeling, as well as biological systems modeling. The language is founded on an explicit distinction between the static and the dynamic views of a system. It introduces thirteen diagram types, grouped into two sets: structural modeling diagrams and behavioral modeling diagrams. UML lacks constructs dedicated to function modeling as such [18], but it provides several built-in mechanisms that allow for an easy extension of the language.

**Fig. 1:**
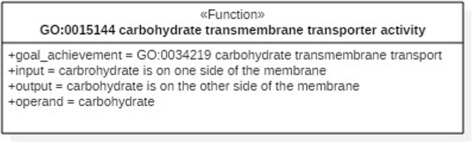
A FueL model of a molecular function, displayed in the compact notation

Among these extension mechanisms there are UML profiles. A *profile* is a light-weight UML mechanism, typically used for extending the language for particular platforms, domains or tasks [11, ch. 12]. It specifies a set of extensions of the UML standard metamodel which include, among others, *stereotypes*. With stereotypes it is possible to extend the standard UML vocabulary with new, specialized model elements. A stereotype can be graphically represented by a dedicated icon, though in the most straightforward form it is represented simply by a stereotype name, surrounded by guillemets and placed above the name of the stereotyped UML element, cf. «Function» in Fig. 1.

We used the profile mechanism for developing a UML extension, called **Fu**nction Mod**e**ling **L**anguage (FueL)^1^, aimed at supporting the modeling of functions, function ascription, and function decomposition. FueL defines 15 stereotypes for representing functions and function structure, as well as 8 stereotypes for modeling function decomposition, subsumption and function dependencies. The full specification of FueL stereotypes is available in [19]. Burek et al. [18] provides a detailed introduction to FueL, based on requirements for function modeling derived from an elaborate review of corresponding literature, in general, as well as of UML modeling constructs related to functions, in particular. In addition to the profile, [18] comprises an axiomatic characterization of the core elements of FueL and discusses its suitability for function modeling with respect to the requirements identified.

In the remainder of the current paper we analyze to which extent FueL can be used for modeling and refactoring MFO. As a prerequisite for this analysis, we begin with a condensed account of FueL.

## Results

### Modeling molecular functions with FueL

**Fig. 2:**
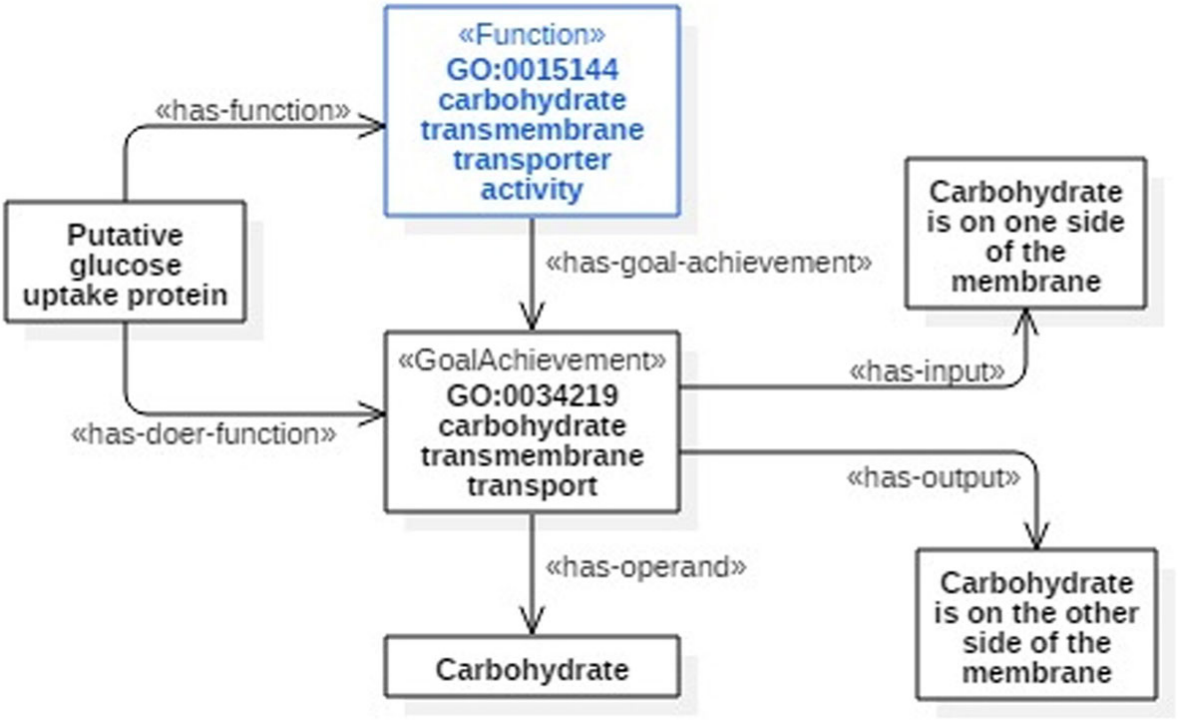
A FueL model of a molecular function, displayed in the extended notation

FueL enables the graphical modeling of functions both in a compact and in an extended form. The compact form is particularly suited for large models containing many functions, whereas the extended form is designed for visualizing the dependencies within the structure of a single function or between several functions. Figures 1 and 2 present an exemplary FueL model, depicting the structure of MFO function GO:0015144: *carbohydrate transmembrane transporter activity*. Figure 1 presents the compact notation, whereas the extended notation is shown in Fig. 2. The stereotypes utilized in the figures are discussed in the remainder of this section.

#### Functions

A function in FueL is understood as a role that an entity plays in the context of some goal achievement, e.g. in a teleological process. Put differently, a role in virtue of which the transition to a goal situation is achieved, or which contributes to such achievement, constitutes a function. An entity, like putative glucose uptake protein in Fig. 2, that plays such a role has that role as its function. This account of functions is similar to [20], where a biological function of a molecule is described as the role that the molecule plays in a biological process. In this sense, the function GO:0015144: *carbohydrate transmembrane transporter activity*, defined in GO as ‘catalysis of the transfer of carbohydrate from one side of the membrane to the other’, depicts the catalyst role in the teleological process of transferring carbohydrate from one side of the membrane to the other.

In terms of the structure we can therefore say that a function specification contains as its part a specification of a goal achievement, understood as a teleological entity which is specified in terms of a transformation from an input situation to an output situation. As presented in Figs. 1 and 2, a function is depicted by a UML classifier with a stereotype «Function». It connects to its goal achievement by an association with a stereotype «has-goal-achievement» in the extended notation, whereas the compact notation utilizes the attribute goal_achievement.

#### Goal achievements

In FueL, a goal achievement (GA) is defined as a teleological transition, i.e., as a transition to a certain output situation (the goal). Note that transitions further exhibit an input situation. The GA characterization applies at both the individual and categorial level. With respect to the latter, input and output are defined as follows:

- The input category *x* of goal achievement *y* is a situation category such that every instance of *y* is a transition starting from a situation instantiating *x*.
- The output *x* of goal achievement *y* is a situation category specifying the situations in which instances of *y* result by transition. Every instance of *y* is a transition resulting in a situation instantiating *x*.

For example, the goal achievement (category) *carbohydrate transmembrane transport* establishes the input category, the instances of which are situations of carbohydrate being on one of the two sides of the membrane, and the output category, the instances of which are situations of carbohydrate being on the other side of the membrane. This means that every instance of *carbohydrate transmembrane transport* exhibits a transition from an instance of the input category to an instance of the output category, i.e. from individual situations of carbohydrate located on one side of the membrane, to individual situations of carbohydrate located on the other side of the membrane.

In the compact notation, the input is captured by the input attribute of a function, see Fig. 1. In contrast, Fig. 2 illustrates that an association with stereotype «has-input» is used for connecting a function with its input in the extended notation. The representation of outputs is analogous in both variants.

Typically, a transformation from an input to an output situation is a process. At the categorial level, the GA can then be understood as a process category. In the running example, the GA is a teleological process category, namely of carbohydrate transfer from one side of the membrane to the other. This process exhibits the causal transition from the situation of carbohydrate being on one side of the membrane to the situation where carbohydrate is on the other side of the membrane.

#### Mode of goal achievement

In some cases the specification of a function is not reduced to a mere input-output pair, but it defines constraints on the method of function realization. For example, the molecular functions GO:0015399: *primary active transmembrane transporter activity* and GO:0015291: *secondary active transmembrane transporter activity* share the same input: solute is on one side of the membrane, and the same output: solute is on the other side of the membrane. Therefore, the pure input-output views of the functions are equal. However, they are distinct due to the way in which they achieve the goal. The former function is realized by means of some primary energy source, for instance, a chemical, electrical or solar source, whereas the latter relies on a uniporter, symporter or antiporter protein. Thus we see that the functions provide the same answer to the question on *what* is to be achieved, however they provide different answers on *how* that is realized. In order to represent this distinction, in FueL we introduce another component of function structure, called *Mode of Goal Achievement* (or Mode of Realization). The mode *x* of the goal achievement *y* specifies the way in which *y* transforms the input to the output situation. For GO:0015399 the mode is: by some primary energy source, for instance chemical, electrical or solar source, and for GO:0015291 it is: by uniporter, symporter or antiporter protein. The mode is a constraint on the function realization, which does not affect the input or the output. For example, if one adds to the function of transmembrane transport the constraint that the transport should be realized by the uniporter protein, then the input and the output remain unchanged. However, the function as such changes in that not every transportation process realizes it, but only those that are driven by a uniporter protein.

#### Participants

Often goal achievements are expressed by action sentences of natural language and thus the results of linguistic analysis of action sentences can be applied to the analysis of the structure of goal achievements. In linguistics, the role that a noun phrase plays with respect to the action or state described by the verb of a sentence is called a thematic role [21]. The specifications of molecular functions in MFO often contain two thematic roles – a patient (called an operand in FueL) and an actor (called a doer in FueL). An operand indicates the entity undergoing the effect of the action. At the categorial level we say that an operand *y* of the goal achievement *x* specifies a category *y* such that instances of *x* operate on instances of *y*. GO:0015144 operates on (transports) carbohydrate.

A doer is not as common in MFO as an operand. For example, in the discussed carbohydrate transmembrane transport function no doer is indicated. Typically, a doer is a part of the GA in cases where the mode of realization is provided. For instance, the functions GO:0015292: *uniporter activity* and GO:0015293: *symporter activity* both specify the mode of realization and each indicates its doer, namely the respective protein.

### Patterns of function subsumption

Behind function subsumption various distinct relations are actually implicitly hidden [14]. In this section we introduce three patterns for function subsumption that can be indicated by FueL stereotypes [19]. The subsequent “Application” section demonstrates the application of those patterns to the modeling of MFO.

In FueL the notion of function subsumption is founded on the subsumption of goal achievements. We say that the function *x* is subsumed by the function *y* if the goal achievement of *x* is subsumed by the goal achievement of *y*. Since goal achievements are quite complex entities, it is not trivial to answer the question of what it means that one goal achievement subsumes another. Here, however, the analysis of GA structure is helpful, which pertains to the intensional aspects of the corresponding GA category, as discussed in previous sections. Based on this approach one can detect various patterns of function subsumption.

#### Operand specialization

**Fig. 3:**
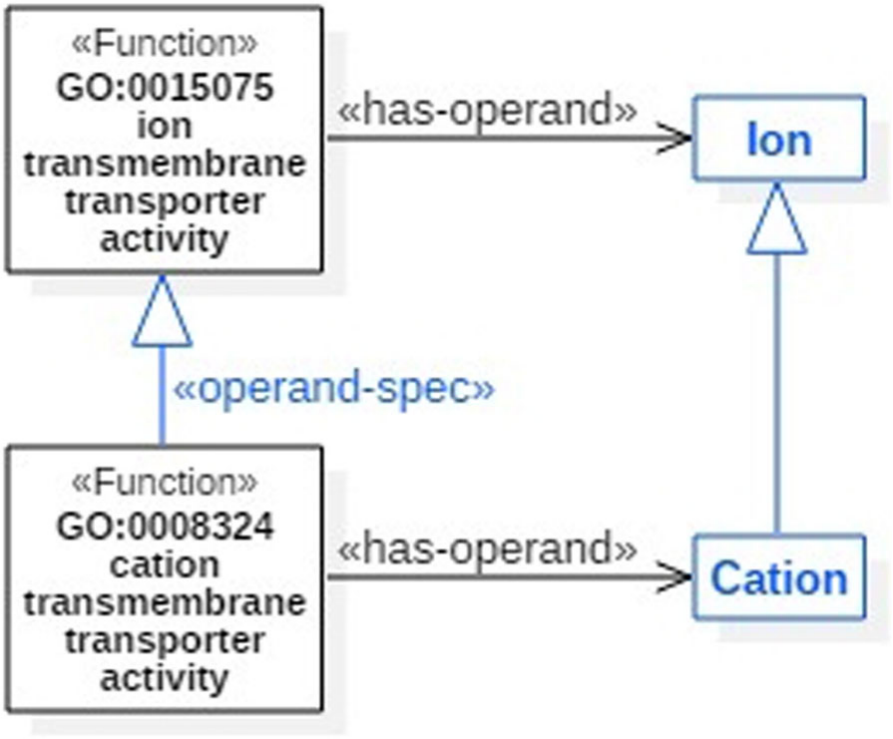
An example of operand specialization

Since function specifications often contain operands, it is very common to construct a hierarchy of functions on the basis of the taxonomic hierarchy of their operands. In fact, this pattern is applied frequently in MFO. Consider, for instance, the functions GO:0015075: *ion transmembrane transporter activity* and GO:0008324: *cation transmembrane transporter activity*, linked by the is_a relation in GO. As presented in Fig. 3 the relation between those two functions is based on the relation of their operands, as cation is subsumed by ion.

Function subsumption by operand specialization is depicted in FueL with a specialization link with the stereotype «operand-spec». The supplier of the link is the subsumed function, the client is the subsumer.

#### Mode addition

**Fig. 4:**
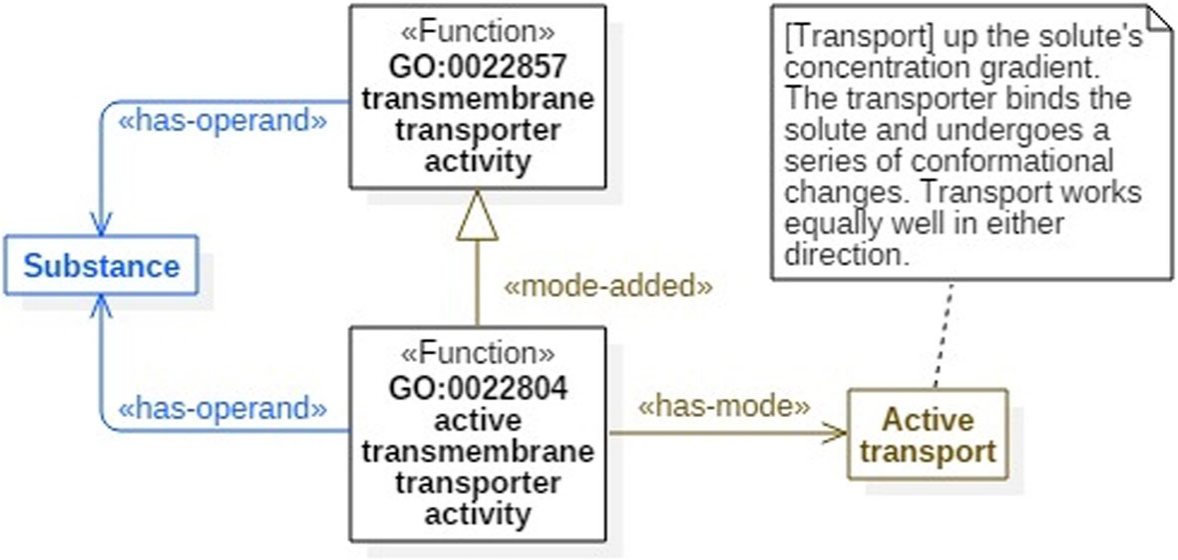
An example of specialization by mode addition

Another pattern of function subsumption, frequently met in MFO, is based on modes of goal achievement. Consider two functions presented in Fig. 4, GO:0022857: *transmembrane transporter activity* and GO:0022804: *active transmembrane transporter activity*. Both share the same operand, namely substance, as well as the same input-output pair – operand is on one side of the membrane and operand is on the other side of the membrane. In this sense those functions are equal. However, they differ in that the former does not define any mode of realization, whereas the latter has the following mode defined: the transporter binding the solute undergoes a series of conformational changes. Therefore, one can say that GO:0022804 specializes GO:0022857 by addition of a mode. We say that function *x* is subsumed by the function *y* by mode addition if *x* is subsumed by *y* and *x* has some mode, whereas *y* has no mode assigned. Function subsumption by mode addition is depicted in FueL by means of a specialization link with stereotype «mode-added». The subsumed function is the supplier of the link and the subsuming function is a client.

#### Mode specialization

Subsumption of functions can be based on the mode of realization also in cases where a parent function has already a mode assigned. Consider, for instance, the function GO:0022804: *active transmembrane transporter activity* having the mode: transporter binds the solute and undergoes a series of conformational changes and the function GO:0015291: *secondary active transmembrane transporter activity* with the mode: transporter binds the solute and undergoes a series of conformational changes driven by chemiosmotic energy sources, including uniport, symport or antiport. The latter clearly characterizes particular modes of active transmembrane transport. Consequently, it seems intuitive to say that GO:0015291 specializes GO:0022804 (as is the case in GO). We call this type of function subsumption the subsumption by mode specialization and define it as follows: The function *x* is subsumed by the function *y* by mode specialization if *x* is subsumed by *y* and mode *r* of *x* specializes mode *s* of *y*. In FueL function subsumption by mode specialization is depicted with a specialization link with stereotype «mode-spec». The subsumed function is the supplier of the link and the specialized function is a client.

### Application

#### Objectives of applying FueL

In general, graphical modeling languages like UML are broadly applied in connection with diverse tasks, such as brainstorming, collaborative design, and the modeling of key principles of systems and subject matters. Another broad area of application concerns standardized visualization, for example, for documentation purposes.

Regarding FueL more specifically, its application to GO and MFO, in particular, pursues three objectives. The first objective is the use of FueL for establishing a semantic basis for molecular functions that supports the representation of functions in a systematic way, beyond their textual description. Moreover, the discussed patterns represent basic knowledge of the interrelations between biological processes and molecular functions. The part_of relation between biological processes and molecular functions can be mapped to the has-goal-achievement association between functions and goal achievements. Figure 2 comprises a corresponding example, where the process GO:0034219: *carbohydrate transmembrane transport* is modeled as a goal achievement of the function GO:0015144: *carbohydrate transmembrane transporter activity*.

**Fig. 5:**
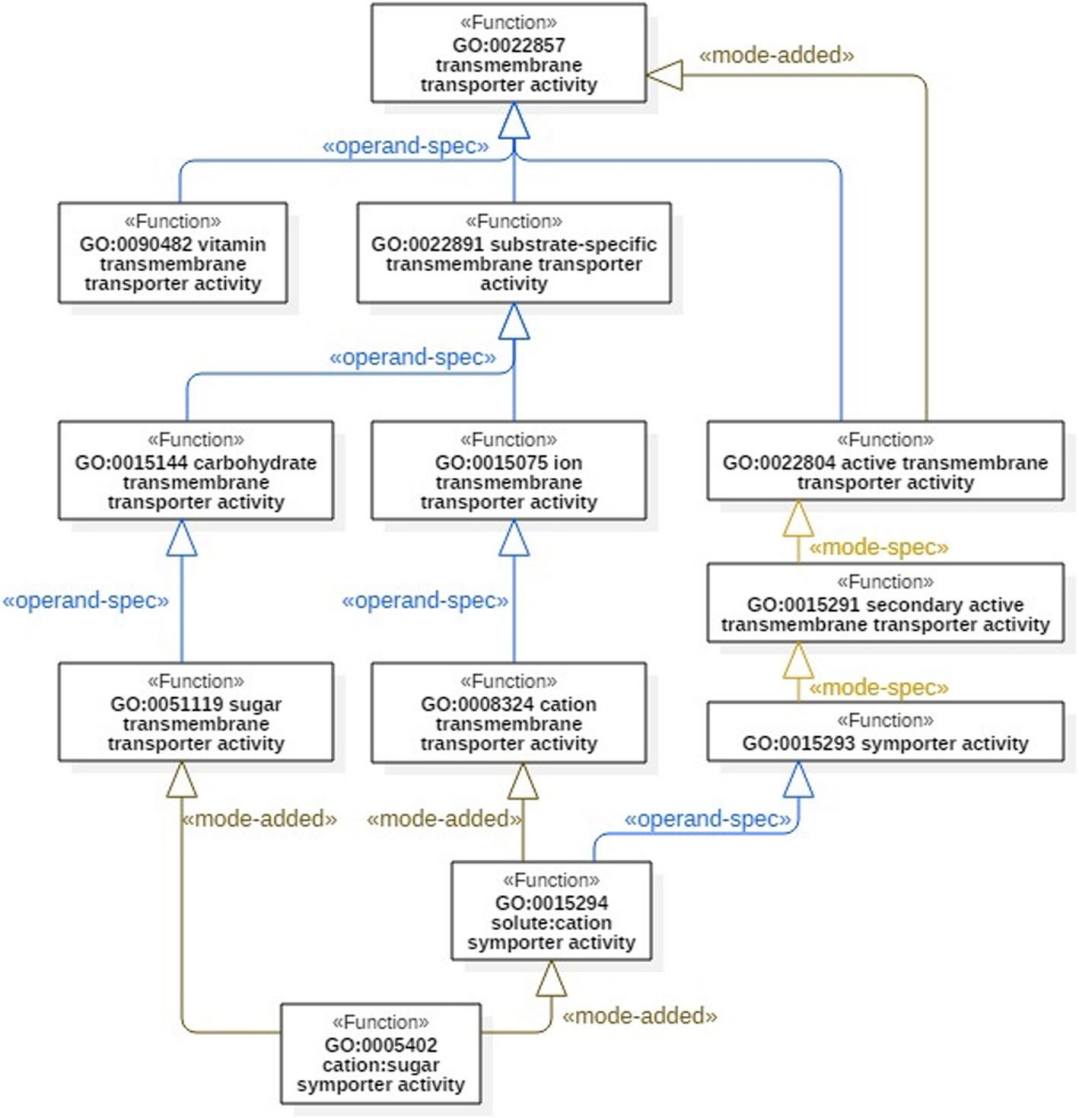
A segment of MFO modeled with FueL

The second and the main objective of applying FueL to MFO is to explicitly document design choices and the subsumption patterns utilized implicitly in MFO. Figure 5 presents such a documentation of a fragment of MFO in terms of FueL. The patterns are indicated by the FueL stereotypes, which enables an easy-to-grasp visualization of the structure of MFO as well as of the underlying design choices. Stereotypes further allow for displaying multiple facets of function subsumption, as in the case of GO:0022804, which can be understood to involve mode addition as well as operand specialization. The explicit specification of design choices makes the ontology much more intelligible for human users, which is a major benefit of this approach.

Thirdly, the application of FueL reveals potential for the refactoring and revision of GO. Contributing to the latter is another important objective of our work. For instance, the application of FueL in modeling the functions GO:0022857: *transmembrane transporter activity* and GO:0022891: *substrate-specific transmembrane transporter activity* shows that both share similar goal achievements: transfer of an operand from one side of a membrane to the other, with input: operand is on one side of the membrane, and output: operand is on the other side of the membrane. Consequently and following FueL, a potential difference between GO:0022857 and GO:0022891 can be searched for in their operands. For GO:0022857 that is ‘a substance’, whereas for GO:0022891 it is ‘a specific substance or group of substances’.

#### Analysis of refactoring options

Let us consider the previous case in greater detail, thereby identifying three possibilities of analyzing and refactoring MFO elements based on FueL. A first FueL view on a selected set of functions that includes the two just named is depicted in Fig. 5. It rests on the assumption that ‘a specific substance or group of substances’ can be considered as a subclass of ‘a substance’. Accordingly, Fig. 5 documents explicitly the pattern of subsumption between GO:0022857 and GO:0022891, namely as a case of operand specialization. The same aspect applies to GO:0022804, the operand of which is also ‘a specific substance or group of substances’.

This straightforward approach, however, may be reconsidered, especially the question of what the actual relation between ‘a substance’ and ‘a specific substance or group of substances’ is. One indication may be derived from GO:0022892: *substrate-specific transporter activity* (not displayed in Fig. 5), which is another parent function of GO:0022891 in MFO. An operand of GO:0022892 is exemplified by macromolecules, small molecules or ions. If we thus interpret ‘a specific substance or group of substances’ as macromolecules, small molecules or ions, this seems to suggest that further functions such as GO:0090482: *vitamin transmembrane transporter activity* and GO:0015238: *drug transmembrane transporter activity* should also be considered as subclasses of substrate-specific transmembrane transporter activity. The latter is currently not the case in MFO, such that positioning those functions under GO:0022891 is a refactoring option, independently of adopting FueL as a representation language. If FueL is employed, these considerations yield an alternative to Fig. 5 (not shown in a separate figure), where, for instance, GO:0090482 is an operand specialization of GO:0022891 instead of GO:0022857. GO:0022804, based on its operand identical to that of GO:0022891, would turn into a specialization of the latter by mode addition.

Another possible refactoring originates from an analysis of the subclasses of GO:0022891: *substrate-specific transmembrane transporter activity*. Examining those subclasses we find that they differ only in their operands. Each of those functions specifies the transport of a specific kind of substance, for example, ion (GO:0015075) or carbohydrate (GO:0015144). This suggests that the distinction between the operands of GO:0022857 and GO:0022891 is only superficial. According to this interpretation, GO:0022891 is merely used for the organization of the function taxonomy, i.e., for grouping all functions that are distinguished by their operands. GO:0022891 would then be a duplication of GO:0022857, which is only introduced into MFO for structuring purposes, but which captures no distinct specification of a biological function. The introduction of such grouping artifacts is a design choice that is clearly not desirable, especially in complex ontologies like MFO or GO overall. One reason for avoiding them is that in many cases of using them subclasses occur after several steps of specialization that do not or not exactly match the grouping specification. For example, GO:0005402: *cation:sugar symporter activity* in Fig. 5 may be questioned to be a (pure) substrate-specific transmembrane transporter activity, given the subsumption path via GO:0022804 involving mode addition and mode specialization.

**Fig. 6:**
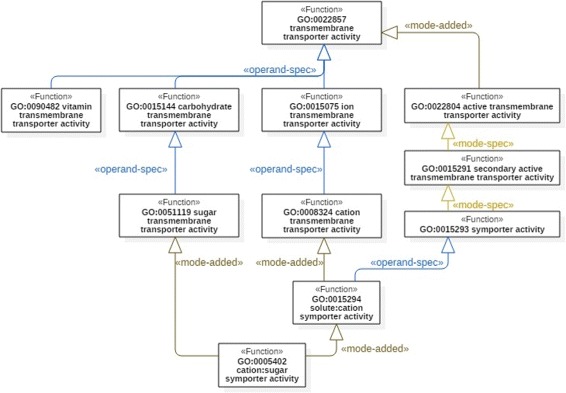
A refactoring of the segment of MFO in Fig. 5

Concerning the purpose of better organization of the taxonomy, we argue that FueL proves beneficial, not at least due to its stereotyped links. As illustrated in Fig. 6, the application of FueL allows for dropping GO:0022891 (if interpreted as a grouping artifact), on the one hand, while on the other hand, FueL enables the explicit specification of design choices by stereotyped specialization links. Note that this supports the “local” grouping of the immediate, explicit subclasses of a given function based on the link stereotypes.

The decision on such refactoring options, as in any modeling enterprise, is the responsibility of the modeler(s), i.e., GO developers in our case. Regarding refactoring means and methods, however, we argue that the above analysis demonstrates how graphical languages such as FueL, similarly as in software and systems engineering, can drive and support the revision of biological ontologies like MFO. Although graphical modeling may not be efficient for representing the complete content of large and complex ontologies, we defend the position that graphical languages can still be extremely helpful, for example, for depicting ontology fragments that exhibit problems. Moreover, in view of ontology development as a collaborative enterprise, graphical modeling formalisms like FueL help to conduct community based analysis in structured ways.

## Discussion

The ideas underlying the structure of functions, introduced in FueL, are the result of an analysis of the current state of the art of function modeling in software, systems and ontological engineering. For instance, the interpretation of a function in terms of a role is common not only in biological systems [20], but also in function modeling in mechanical engineering [22–24].

The notion of goal achievement grasps the teleological character of a function, its orientation towards some goal. This aspect is stressed in many approaches to function representation, e.g. [25–27]. In particular, defining a function in terms of input-output pairs is present in modeling technical artifacts [28, 29]. The mode of realization, also called the way-of-function-achievement, which specifies constraints on the method of how a function is realized, can be found in [30], among others.

To the best of our knowledge, the presented patterns of function decomposition are not collected and integrated into any other single modeling framework, though the techniques themselves are commonly used, especially in software and systems engineering, e.g. see the function-means-context link in [31] or the decomposition with zig-zaging in [32].

Another aspect worth of discussion is the practical applicability of the proposed approach, in particular, with respect to GO and its Molecular Function Ontology. In this connection it appears realistic to admit that the mere existence of FueL as a UML profile does not render the approach ready for an immediate, production-level adoption in the day-to-day curation of function terms in MFO. The tool set capable of handling MFO (and of GO overall), for example, in terms of its size and in accordance with its recent turn to its representation in OWL exhibits basically no connection to the world of UML and corresponding modeling tools. Insofar the direct application of FueL involves bridging this gap manually, which is limiting to small-scale, focused case analyses at the present stage.

Nevertheless, we think that the detailed discussion of refactoring options in the previous section illustrates the utility of such analyses. There is a significant potential in view of the fact that, clearly, many more exemplary or specific cases can and should be made based on MFO. For instance, analyzing the terms GO:0016209: *antioxidant activity* and GO:0003824: *catalytic activity* together with their subclasses systematically, some of which they share, one may raise the question of why GO:0004601: *peroxidase activity* specializes antioxidant activity, but is not subsumed by *catalytic activity*, despite the definition of GO:0004601, which starts with ‘Catalysis of reaction: donor + [...]’. Moreover, there are various groups of terms of the form *X* *regulator activity*, *X* *activator activity* and *X* *inhibitor activity*, at different levels of generality (e.g., cf. *receptor* vs. *acetylcholine receptor* for *X*). Such groups may justify a novel, common pattern of function subsumption, namely based on the output of the corresponding goal achievement. One further finds that goal achievements are not yet present in GO in a number of cases, i.e., there are no processes corresponding to available functions.

Further analysis of MFO terms on the basis of FueL constituents such as operands and modes leads to the identification of functions that are specialized (1) almost exclusively by mode additions or mode specializations, whereas the subclasses of others (2) primarily rely on operand specialization. GO:0009055: *electron carrier activity* may serve as an example of the former case. At least seven out of its eight direct is_a children clearly arise through mode addition or specialization, e.g., GO:0045154: *electron transporter, transferring electrons within cytochrome c oxidase complex activity* and GO:0045156: *electron transporter, transferring electrons within the cyclic electron transport pathway of photosynthesis activity*. In contrast, GO:0004872: *receptor activity* has seven direct subclasses (*apolipoprotein*, *cargo*, GO:0005055: *laminin*, *pattern recognition*, GO:0038023: *signaling*, GO:0099600: *transmembrane* and *virus receptor activity*), the subsumption links to which involve operand specialization (and only *cargo receptor activity* a mode addition, as well).

Besides such distinctions of the way in which a term relates to its overall set of direct subclasses, we observe that FueL-guided analysis can generally contribute to comparing terms and their definitions more easily. This applies in particular cases, e.g., when wondering about the (in)difference between the operand *signal* of GO:0038023 and operand *extracellular or intracellular signal* of GO:0099600. A decision on this question supports the comparison of the overall definitions of both terms. Further considerations may be concerned with a more general perspective. Looking at the operands identified in our analyses, we find that some operands are named by role terms such as *messenger* (w.r.t. GO:0004872), others have non-role names, e.g. *laminin* (w.r.t. GO:0005055), and yet others mix both aspects, like *hydrogen or electron acceptor* in GO:0016491: *oxidoreductase activity*. This yields a connecting factor to the field of roles and role analysis, cf. e.g. [33–35], which may lead to novel refactoring considerations for MFO as well as to future refinements of function subsumption patterns.

Overall, on the one hand we do see significant potential based on inspecting MFO manually in a systematic and structured way, using FueL. On the other hand, the purely manual approach is a limitation at the present stage and hampers an extensive evaluation, which would ideally involve direct participation by GO developers.

Despite the shortcoming regarding validation in practice, we argue that presenting and demonstrating our approach in a biological context is already beneficial. The aspect of applying it systematically to specific function terms, which may also be conducted merely on the conceptual basis of FueL, almost independently of the UML language aspects, is elaborated above. But more can be said. First, although we consider MFO as a major case of interest, FueL is applicable to functions in arbitrary domains and contexts, cf. [18]. The approach presented may therefore be of interest concerning functions covered in other biomedical ontologies. Secondly, we see many routes of future work that can be pursued, possibly in collaboration with other groups. In the context of MFO, there are at least the ideas (1) to provide tools that support the use of FueL by ontology developers and augment an established ontology lifecycle, as well as (2) to develop (semi-)automated approaches and software that can be applied to the existing MFO structure, for example, in order to determine instances of subsumption patterns. This leads to a final point here, though of no less importance, where subsumption patterns are a natural candidate to deal with. Given OWL as the current basis of development and reasoning of many biomedical ontologies, a way to bridge between OWL and FueL is highly desirable, or – at the very least – the transfer of FueL-based function analysis and representation to a corresponding use of OWL. We expect either task to be ambitious. FueL is equipped with a formalization in first-order logic [18], which must be respected and related to clearly if an OWL formalization or translation is derived from FueL. Another issue along similar lines is the treatment of UML stereotypes in OWL, as these are meta-classes in UML. There are a number of conceivable options to tackle their treatment in OWL, ranging from not making them explicit over the use of punning or annotations [36] to using multiple OWL ontologies for one FueL model. Identifying pros and cons of such options with respect to particular purposes in the context of biomedical ontologies remains an interesting future effort.

## Conclusions

In the current paper we present and discuss applications of UML and patterns of function subsumption to the modeling and refactoring of biological ontologies. In particular, we developed a UML profile for function modeling, called the **Fu**nction Mod**e**ling **L**anguage (FueL) [19], and apply it to the modeling and refactoring of segments of the Molecular Function Ontology.

The application of FueL enables the systematic, graphical representation of functions and thereby of information that is currently available in MFO mainly in the form of textual descriptions. We elaborate that behind the extensional is_a relation, which is used for the construction of MFO, several different patterns of intensional subsumption can be determined. Modeling MFO via FueL helps in identifying pattern instances that occur implicitly in MFO. Moreover, FueL provides the means of referring to those patterns directly in the hierarchy of molecular functions. We argue that this can help in making the ontology structure more comprehensible for human users and that it supports communication. The claim is demonstrated by an analysis and a model of an MFO fragment with FueL, from which we derive several refactoring options.

Besides proposing the adoption of FueL and the particular refactoring options in this paper, for future work we consider first the continued analysis of MFO. Extending this to a larger scale may require establishing software support, e.g., for identifying subsumption pattern instances within MFO (semi-)automatically. Moreover, FueL and its methods may also be transferred to or may yield new methods for common languages of biomedical ontologies, nowadays including OWL.

## Endnote

^1^ In contrast to ‘FuML’ in a preceding publication [37] (cf. also the Acknowledgments section below), the acronym ‘FueL’ has been adopted for a better terminological distinction from other efforts, like fUML [38] by OMG.
